# Exploring Factors Associated with Women’s Willingness to Provide Digital Fingerprints in Accessing Healthcare Services: A Cross-Sectional Study in Urban Slums of Bangladesh

**DOI:** 10.3390/ijerph19010040

**Published:** 2021-12-21

**Authors:** Sabuj Kanti Mistry, Fahmida Akter, Md. Belal Hossain, Md. Nazmul Huda, Nafis Md. Irfan, Uday Narayan Yadav, Daniel M. L. Storisteanu, Amit Arora

**Affiliations:** 1 BRAC James P Grant School of Public Health, BRAC University, Dhaka 1212, Bangladesh; smitra411@gmail.com (S.K.M.); fahmida.akter@bracu.ac.bd (F.A.); belal.hossain@ubc.ca (M.B.H.); 2 Centre for Primary Health Care and Equity, University of New South Wales, Sydney, NSW 2052, Australia; 3 Department of Health Research, ARCED Foundation, 13/1, Pallabi, Mirpur-12, Dhaka 1216, Bangladesh; 4 Department of Public Health, Daffodil International University, Dhaka 1207, Bangladesh; 5 School of Population and Public Health, University of British Columbia, Vancouver, BC V6T 1Z3, Canada; 6 School of Health Sciences, Western Sydney University, Campbeltown, NSW 2560, Australia; hudasoc2020@gmail.com; 7 School of Population Health, University of New South Wales, Sydney, NSW 2052, Australia; 8 Institute of Nutrition and Food Science, University of Dhaka, Dhaka 1000, Bangladesh; nafis.irfan35@gmail.com; 9 Interdisciplinary Graduate Program in Human Toxicology, University of Iowa, Iowa City, IA 52242, USA; 10 National Centre for Epidemiology and Population Health, Research School of Population Health, The Australian National University, Canberra, ACT 0200, Australia; unyadav1@gmail.com; 11 Laboratory of Viral Zoonotics, University of Cambridge, Cambridge CB3 0ES, UK; danstori@gmail.com; 12 Translational Health Research Institute, Campbelltown Campus, Western Sydney University, Penrith, NSW 2751, Australia; 13 School of Health Sciences, Western Sydney University, Campbelltown Campus, Penrith, NSW 2751, Australia; 14 Oral Health Services, Sydney Local Health District and Sydney Dental Hospital, NSW Health, Surry Hills, NSW 2010, Australia; 15 Discipline of Child and Adolescent Health, Sydney Medical School, Faculty of Medicine and Health, The University of Sydney, Westmead, NSW 2145, Australia; 16 Health Equity Laboratory, Campbelltown, NSW 2560, Australia

**Keywords:** Bangladesh, digital fingerprints, access, health service use, slums, social disadvantage

## Abstract

Digital fingerprints are increasingly used for patient care and treatment delivery, health system monitoring and evaluation, and maintaining data integrity during health research. Yet, no evidence exists about the use of fingerprinting technologies in maternal healthcare services in urban slum contexts, globally. The present study aimed to explore the recently delivered women’s willingness to give digital fingerprints to community health workers to access healthcare services in the urban slums of Bangladesh and identify the associated factors. Employing a two-stage cluster random sampling procedure, we chose 458 recently delivered women from eight randomly selected urban slums of Dhaka city, Bangladesh. Chi-square tests were performed for descriptive analyses, and binary logistic regression analyses were performed to explore the factors associated with willingness to provide fingerprints. Overall, 78% of the participants reported that they were willing to provide digital fingerprints if that eased access to healthcare services. After adjusting for potential confounders, the sex of the household head, family type, and household wealth status were significantly associated with the willingness to provide fingerprints to access healthcare services. The study highlighted the potentials of using fingerprints for making healthcare services accessible. Focus is needed for female-headed households, women from poor families, and engaging husbands and in-laws in mobile health programs.

## 1. Introduction

The global healthcare system has moved away from relying on paper-based record-keeping towards increasingly digital health records to augment its efficiency in resource usages and health service quality [1]. Harnessing optimum benefits from digital platforms requires accurate identification of the patient/client. From both the healthcare providers’ and care recipients’ perspectives, unique identification of the patients/clients is crucial on both the healthcare-provider side and care-recipient side in any healthcare settings (e.g., such as community or hospital/clinics). This is important for the smooth delivery of patient/client care and treatment, health system monitoring and evaluation, and maintaining data integrity during health research [2]. Moreover, unique patient identification can help healthcare workers to improve the quality of health services and minimize the errors caused due to misidentification and misclassification [3,4]. Therefore, digital health records are of crucial importance for healthcare management [5].

Biographic-based parameters (e.g., patient names, birth dates, national identification or social security numbers, clients’ addresses) are commonly used for patient/client identification. However, in resource-poor settings, these types of identification systems can cause mistakes due to inconsistent spelling of names or addresses, ambiguity about birth dates, frequently changing phone numbers and addresses, and deliberate avoidance of being identified due to social stigma [2]. The situation is considerably worse in the fast-growing unplanned urban slums in resource-constrained countries (e.g., Bangladesh and Kenya), where migration from both rural to urban and within different slums are frequent [6,7,8]. For example, the migration rate is relatively higher (≈50%) in slum areas of Dhaka city (the capital of Bangladesh), compared with other cities of the country [6]. The Bangladesh Bureau of Statistics (BBS) defines slums as “a cluster of compact settlements of 5 or more households which generally grow very unsystematically and haphazardly in an unhealthy condition and atmosphere on government and private vacant land” [9]. In 2014, 13,938 slums were counted in Bangladesh [10]. Patient identification and verification remain highly challenging due to the volatile nature of the urban slums, their higher migration, slum evictions and the marginalization of destitute segments [11]. To overcome this situation, a more reliable and definitive form of identification, such as fingerprint biometrics, could help ensure that care is provided to the right people, leading to a safer and more effective healthcare system in resource-constrained settings [2].

Biometric identification methods are usually developed based on individuals’ biologically unique traits, such as fingerprints, voice, facial structure, iris geometry etc. [12]. The fingerprint is the most widely used biometric identifier [13,14] due to the wide availability of well-developed, portable, and low-cost technologies for digitally capturing fingerprints [12,15]. Research has demonstrated that fingerprinting is highly sensitive and specific for verification [16]. Several studies reported that fingerprint biometrics are feasible and acceptable for accessing healthcare services [17,18]. Yet, limited evidence exists about the use of fingerprinting technologies in health services in urban slum contexts. globally.

To integrate mobile fingerprint scanners into the existing android mHealth app, used for health services via the *Manoshi* program in urban slums in 2007 [11,19], Building Resources Across Communities (BRAC) Bangladesh explored the potential of using Simprints’ low-cost fingerprint technology. *Manoshi* denotes mother, neonate, and child in Bengali. The intervention is a community-based essential healthcare package aimed at reducing morbidity and mortality among mothers, new-borns, and children residing in selected urban slums in Bangladesh by providing maternal and childcare services [11]. The intervention helps create demand for maternal and child healthcare services through behaviour change communication (BCC) and assists in accessing emergency obstetric care services by promoting effective referral linkages. BRAC’s frontline community health workers (CHWs) facilitate delivering BCC interventions among slum dwellers through door-to-door visits, contributing to making the referral linkage functional for ensuring their proper maternal and child health services [16]. The fingerprinting system used consisted of a small, handheld scanner that connects via Bluetooth to the smartphone used by CHWs without a consistent internet connection [20]. A proprietary algorithm using minutia, which are specific points on a fingerprint such as a ridge ending or bifurcation, was used to register and later identify patients. Minutia-based systems are a standard approach to biometric fingerprinting (e.g., [21,22]). This system is in the process of being piloted by BRAC.

In the current paper, we aimed to explore the willingness of the beneficiaries to provide digital fingerprints to BRAC’s CHWs in accessing healthcare services by the slum dwellers of Dhaka city, Bangladesh and to identify the socioeconomic factors associated with the willingness to provide fingerprints in accessing healthcare services. The findings will be beneficial during planning to implement this type of technology in similar contexts by providing information on the determinants to consider.

## 2. Materials and Methods

### 2.1. Study Design and Participants

The study followed a cross sectional-design. We conducted the survey between May and June 2018 among the women who gave birth in the last 12 months preceding the survey. A two-stage cluster random sampling approach was employed to select 458 participants from Dhaka city’s eight randomly selected urban slums. The sample size calculation and the details of the study design can be found elsewhere [11].

### 2.2. Data Collection Tools and Techniques

A pre-tested semi-structured questionnaire was used to collect the information through face-to-face interviews of the study participants. Data collection was accomplished by female research assistants who were recruited based on educational qualification and previous experience with administering maternal and child health surveys. The research assistants were trained extensively on the study’s goal and objectives and every aspect of the questionnaire before the data collection.

### 2.3. Outcome Measure

A digital fingerprint was described to the participants as a technology called digital fingerprinting that does not require ink. Participants were asked if they would be willing to give their digital fingerprints to CHWs to make it easier to receive healthcare services. If not, they were also asked why they do not want to provide digital fingerprints to community health workers for accessing healthcare services.

### 2.4. Explanatory Variables

An extensive review of available studies guided the selection of explanatory variables [23,24,25,26]. The explanatory factors considered in this study were respondents’ age (15–19, 20–24, 25–29, 30–34, and 35+ years), religion (Muslim/other), literacy (yes/no), education level (no education, grade 1–4, 5–9, and 10 or higher), involvement in income-generating activities (yes/no), husband’s age (<25, 25–29, 30–34, 35–39, 40+), husband’s literacy (yes/no), husband’s education level (no education, grade 1–4, 5–9, and 10 or higher), husband’s occupation (business, labourers, service and others), household size (≤4/>4), family types (nuclear/extended), household head (male/female), family income in USD (<120, 120–239, 240–359, 360+), household wealth status (low, moderate, high), and ownership of a mobile phone (yes/no).

The wealth index of an individual household was constructed based on factor analysis [27,28] of key socioeconomic variables: wall types, floor, and roof of the house; ownership of a radio, television, computer, bicycle, mobile phone/telephone, refrigerator, wardrobe, table, chair, watch/clock, bed, sewing machine, bicycle, motor vehicle, or livestock, and access to solar power and electricity. Access to a mobile phone within a family was defined as having a mobile of her own, or any of her family members having a mobile phone she could access.

### 2.5. Statitical Analysis

All analyses properly incorporated clusters (i.e., slums) variation to account for the complex survey design. The distribution of the variables was assessed through descriptive analysis. The Chi-square test was performed to compare women’s willingness to provide digital fingerprints to community health workers for healthcare access with different categories of a variable at a 95% level of significance. We used a design-based binary logistic regression model to determine the association of willingness to provide fingerprints to community health workers for accessing healthcare with different demographic and socioeconomic factors. The final model includes only the variables having *p* < 0.25 in the unadjusted analysis [29]. Both unadjusted and adjusted odds ratios (ORs) were reported with a 95% confidence interval (95% CI). Both unadjusted and adjusted odds ratios (ORs) were reported with a 95% confidence interval (95% CI). All analyses were performed using the statistical software package Stata (Version 13.0) (StataCorp, College Station, TX, USA).

## 3. Results

### 3.1. Characteristics of the Participants

Table 1 shows the summary statistics of the study participants. Of the 458 participants, 14.2% were aged 15–19 years, and 31.7% were 20–34 years. Around 65% of the study participants were literate, 9.2% completed secondary or higher education, and only 16.6% participated in income-generating activities. Approximately 37.1% of the participants’ husbands were illiterate, 11.6% of participants had completed secondary or higher education, and 41.3% were day laborers. Additionally, the number of household members was less than four in most households (62.0%), and most participants had nuclear families (82.0%).

### 3.2. Women’s Willingness to Provide Digital Fingerprints to Access Healthcare Services

Table 2 shows the willingness to provide digital fingerprints to access healthcare services by the participants. Approximately 69% of the participants had heard of digital fingerprints, while 78% were willing to provide digital fingerprints to community health workers for accessing healthcare services. Participants who said they would be unwilling to use fingerprinting mentioned several reasons, including the necessity for their family’s permission (58.9%), that it did not feel necessary (17.8%), they were concerned about the misuse or abuse of their fingerprint record (10%), and that it would take too much time (6.7%).

### 3.3. Factors Associated with Women’s Willingness to Provide Digital Fingerprints

Table 3 shows the bivariate analysis of sociodemographic characteristics and women’s willingness to provide digital fingerprints to community health workers. We found statistically significant associations between women’s willingness to provide digital fingerprints and mobile phone ownership, wealth status, monthly family income, and family type. The participants were willing to provide digital fingerprints if they owned a mobile phone (84.7%), had high wealth status (88.4%), had a family income of 240–359 USD/month (81.6%), or belonged to a nuclear family (82.8%).

The crude and adjusted analysis to explore the factors associated with willingness to provide digital fingerprints are presented in Table 4. The crude analysis showed that the sex of the household head, type of family, household wealth status, and mobile phone ownership were significantly associated with women’s willingness to provide digital fingerprints. After adjusting the model with potential confounders, the sex of the household head, family type, and household wealth status remained as independent predictors for willingness to provide digital fingerprints. In the adjusted model, the participants from nuclear households had 2.8-times-higher odds of willingness to give fingerprints than those from extended families (AOR: 2.83, 95% CI: 1.52–5.24), and the odds were 2.7-times higher when the household head was male rather than female (AOR: 2.65, 95% CI: 0.95–7.34). Additionally, the participants with high wealth status had nearly three-times-higher odds of willingness to provide digital fingerprints than those with low wealth status (AOR: 2.96, 95% CI: 1.63–5.39).

## 4. Discussion

The present study explored recently delivered women’s willingness to give digital fingerprints to community health workers and identified the factors associated with accessing healthcare services in the urban slums of Bangladesh, where BRAC has been implementing its *Manoshi* intervention. Our study found that around eight in ten women were willing to provide fingerprints to community health workers, suggesting that digital fingerprints may be a useful identifier to facilitate women who recently gave birth to access maternal and child healthcare services in urban slums in Bangladesh. Yet, we found that around one-quarter of the participants had not heard of the term ‘digital fingerprint’. This implies that there is still a need for further advocacy through various communication channels if this biometric is to take a more critical role in identification and mobile health in urban slum settings.

Our findings also revealed that many women (around 20%) in slum areas were uninterested in providing digital fingerprints to community health workers to access healthcare services in urban slums. They identified the requirement of family permission, fear of digital fingerprint misuse, and lengthiness of the process as factors for not using digital fingerprints to access healthcare services. The fact that women require their family’s permission may be due to the inherent male-dominated cultural contexts of Bangladesh, where married women’s use of mobile phones and access to the internet are restricted by their husbands [30]. Many women are primarily dependent on male household members who earn money, run their households, and make decisions on financial matters. Women have limited decision-making power within the family regarding utilizing maternal and child health services [25]. As in Bangladesh, in Nepal and India, women’s healthcare decisions are often made without their active participation in the decision making [31]. Nine women in this study cited that the use of digital fingerprints may be a threat to their security and safety, as it might disclose their crucial personal information and can harm their private life, with similar concerns found elsewhere [32]. In the implementation of any biometric system, privacy should be viewed as a universal good to be protected, and substantial technical security measures (e.g., encrypting data when not in use) are vital [33]. All these concerns should be addressed when designing and implementing biometrically enabled mHealth services.

We found that young women (25–29 years) were more willing to provide digital fingerprints than older ones. We did not find any other study on women who gave birth to children in urban slums in Bangladesh or other countries to compare this finding with. The possible reasons are that young women may be more educated and are more likely to use mobile phones for seeking maternal and child health services than older women [24]. A study in rural Bangladesh reported that seeking health services using mobile phones increased with increased years of schooling in both men and women [26]. We also found that both women and their husbands’ educational levels were associated with women’s willingness to provide digital fingerprints. This is reasonably expected, as educated women are more aware of maternal health issues than uneducated women [34]. Our study’s findings suggest that more focus should be given to older and uneducated women to increase their participation in mHealth interventions related to maternal and child health services.

Our study reports that the participants with small household sizes (<4) had a higher willingness to provide fingerprints for accessing healthcare services than those with large household sizes. This may be because women with increased household size are often overburdened with household chores and responsibilities [35], limiting them to seek appropriate and adequate healthcare services using digital fingerprints [11]. Similarly, a study in Dhaka’s slum areas indicated a decreased tendency among mothers to seek childcare services using fingerprints and mobile phones [24]. Evidence shows that males’ active participation in household and childcare activities may prompt women to seek appropriate healthcare services and produce better health outcomes [36,37]. However, male participation in household and childcare activities has been limited in resource-limited countries such as Bangladesh [38,39]. Therefore, it is crucial to initiate campaigns for engaging males in household activities to facilitate women’s participation in maternal and child healthcare services using digital fingerprints.

Consistent with a study in Bangladesh [24], our study found that women with high wealth status and who had mobile phones were more willing to provide digital fingerprints than those with limited wealth and who did not own mobile phones. These findings underpin the fact that economic condition is a major factor in the ownership and use of mobile phones, enhancing women’s participation in healthcare services [11,26] facilitated by mHealth technology. However, women’s ownership of mobile phones is still relatively low in Bangladesh [26]. There is also a gap in women’s mobile phone ownership in terms of their socioeconomic conditions and residency status (rural areas, urban areas and slums) [24,26]. Thus, to enhance the ownership of women’s mobile phones, it might be worthwhile to finance and lend microcredit on zero or at a minimum payable interest to poor women in order that they may access maternal and child health services in urban slums in Bangladesh.

### 4.1. Strength and Limitations

To the best of our knowledge, this is the first study looking at the factors associated with the recently delivered women’s willingness to provide digital fingerprints for accessing healthcare services in urban slums of Bangladesh. Our study adds to the limited international literature exploring individuals’ readiness to provide digital fingerprints to community health workers to access healthcare services and related factors [23,24,26]. However, our study is also subjected to some limitations that need to be considered in interpreting the result. The study was cross-sectional in nature. Therefore, causality cannot be established. Secondly, data were collected using self-reports and thus could be subjected to recall bias. However, we carefully designed the research questions, administered a pre-tested structured questionnaire, recruited skilled interviewers, and employed a multilayer monitoring system to reduce the impact of reporting bias. In addition, most of the factors for women’s willingness to provide digital fingerprints have a straightforward definition. Thus, they are less likely to suffer from differential misclassification due to recall bias across women’s willingness versus unwillingness to provide digital fingerprints. Still, the estimated association could be away from the null for the binary factors and either away from the null or towards the null for the categorical factors. Thirdly, we did not conduct any qualitative research to complement our quantitative findings and provide an in-depth investigation regarding the reasons for willingness or unwillingness to give digital fingerprints. Lastly, our study population is only representative of the slum population in Dhaka city, so that our findings are confined to only this population. Hence, the findings of this study may be only generalizable with a population in a similar setting but may not be generalizable to the slum population in other regions or counties. Future longitudinal studies with a large sample size are required to explore the multi-dimensional risk factors for willingness to give fingerprints in accessing health services over time. Another arena for further research would be exploring the experience of delivering fingerprints in accessing health services applying qualitative methods. This would provide a better understanding of women’s readiness to provide fingerprints in accessing health services and associated factors.

### 4.2. Implications for Policy and Practice

The present study pointed to the potential of employing fingerprints in providing healthcare services among slum dwellers, which is of utmost importance for policy makers and public health practitioners in Bangladesh. The study finding is also particularly significant as the Government of Bangladesh inaugurated the Digital Health Strategy of Bangladesh and put forward the importance of mobile health in improving access to healthcare services. When using fingerprints in providing healthcare services, it is vital to engage family members in mobile health programs, strengthening the existing cyber security and mobile network to remove the barriers to and taboos of providing digital fingerprints. It is also important to deliver mobile text-messaging content using a more user-friendly approach.

## 5. Conclusions

Overall, we found that 78% of the participants were willing to provide digital fingerprints if that eased their access to healthcare services in urban slums in Dhaka city. Participants with nuclear households, male-headed families, and those with higher wealth status tended to be more willing to provide fingerprints to access healthcare services. The study highlighted the potential of using fingerprints for making healthcare services accessible to women who had recently given birth in urban slums in Dhaka. However, misconceptions and fear need to be addressed before designing and implementing mHealth services in slum areas in Bangladesh. Special focus is needed for female-headed households, those from low-income families, and in engaging their husbands in mobile health programs.

## Figures and Tables

**Table 1:** Background characteristics of the respondents (N = 458).

Characteristics	n	%
Characteristics of Participants		
Age (years)		
15–19	65	14.2
20–24	145	31.7
25–29	138	30.1
30–34	74	16.2
35+	36	7.9
Religion		
Muslim	451	98.5
Other ^1^	7	1.5
Literacy (Can Read and Write)		
yes	294	64.2
no	164	35.8
Level of Education		
no education	77	16.8
primary incomplete ^2^	105	22.9
primary or secondary incomplete ^3^	234	51.1
secondary or higher ^4^	42	9.2
Involved in Income-Generating Activities		
yes	76	16.6
no	382	83.4
Characteristics of Their Husband		
age (years)		
<25	38	8.3
25–29	144	31.4
30–34	103	22.5
35–39	122	26.6
40+	51	11.1
Literacy (Can Read and Write)		
yes	288	62.9
no	170	37.1
Level of Education		
no education	104	22.7
primary incomplete ^2^	77	16.8
primary or secondary incomplete ^3^	224	48.9
secondary or higher ^4^	53	11.6
Current Occupation		
business	127	27.7
labourer	189	41.3
regular job	128	28.0
others	14	3.1
Household Characteristics		
Sex of Household Head		
male	439	95.9
female	19	4.2
household size		
≤4	285	62.2
>4	173	37.8
Type of Family		
nuclear	377	82.3
extended	81	17.7
Wealth Status		
low	126	27.5
middle	89	19.4
high	243	53.1

^1^ Hinduism, Christianity, Buddhism; ^2^ primary incomplete = completed grade 1–4; ^3^ primary or secondary incomplete = completed grade 5–9; ^4^ secondary or higher = completed grade 10 or higher.

**Table 2:** Willingness to provide digital fingerprints to access healthcare services (N = 458).

Characteristics	n	%
Heard About Digital Fingerprints		
yes	316	69.0
no	142	31.0
Willingness to Provide Digital Fingerprints to Access Healthcare		
yes	359	78.4
no	99	21.6
Willingness to Provide Digital Fingerprints		
yes	368	80.4
no	90	19.7
Reasons for Unwillingness to Provide Fingerprints		
do not feel it is necessary	16	17.8
it might be misused or abused	9	10.0
it would take too much time	6	6.7
family permission required	53	58.9
other	6	6.7

**Table 3:** Bivariate analysis of sociodemographic characteristics of women and their willingness to provide digital fingerprints (N = 458).

Characteristics	Women’s Willingness to Provide Digital Fingerprints		
	No	Yes	*p* Value
	n (%)	n (%)	
Women’s Characteristics			
Age (years)			
15–19	15(23.1)	50(76.9)	0.466
20–24	30(20.7)	115(79.3)	
25–29	20(14.5)	118(85.5)	
30–34	17(23.1)	57(76.9)	
35+	23(22.2)	77(77.8)	
Religion			
Muslim	90(20.0)	361(80.0)	0.187
Other ^1^	0(0.0)	7(100)	
Literacy (Can Read and Write)			
yes	52(17.7)	242(82.3)	0.157
no	38(23.2)	126(76.8)	
Level of Education			
no education	21(27.3)	56(72.7)	0.065
primary incomplete ^2^	22(21.0)	83(79.1)	
primary or secondary incomplete ^3^	44(18.8)	190(81.2)	
secondary or higher ^4^	3(7.1)	39(92.9)	
Involved in Income-Generating Activities			
yes	20(26.3)	56(73.7)	0.109
no	70(18.3)	312(81.7)	
Husband Characteristics			
Age (years)			
<25	8(21.1)	30(79.0)	0.639
25–29	31(21.5)	113(78.5)	
30–34	22(21.4)	81(78.6)	
35–39	18(14.8)	104(85.3)	
40+	11(21.6)	40(78.4)	
Literacy (Can Read and Write)			
yes	51(17.7)	237(82.3)	0.173
no	39(22.9)	131(77.1)	
Level of Education			
no education	27(26.0)	77(74.0)	0.098
primary incomplete	16(20.8)	61(79.2)	
primary or secondary incomplete	42(18.8)	182(81.3)	
secondary or higher	5(9.4)	48(90.6)	
Occupation			
business	22(17.3)	105(82.7)	0.489
labourer	42(22.2)	147(77.8)	
regular job	22(17.2)	106(82.8)	
other	4(28.6)	10(71.4)	
Household Characteristics			
Sex of Household Head			
male	82(18.7)	357(81.3)	0.012
female	8(42.1)	11(57.9)	
Household Size			
≤4	52(18.3)	233(81.8)	0.331
>4	38(22.0)	135(78.0)	
Type of Family			
nuclear	65(17.2)	312(82.8)	0.005
extended	25(30.9)	56(69.1)	
Family Income Per Month (USD)			
<120	30(33.3)	60(66.7)	0.011
120–239	126(29.2)	306(70.8)	
240–359	30(18.4)	133(81.6)	
360+	24(20.9)	91(79.1)	
Wealth Status			
low	40(31.8)	86(68.3)	0.000
middle	22(24.7)	67(75.3)	
high	28(11.5)	215(88.4)	
Ownership of Mobile Phone			
yes	38(15.3)	211(84.7)	0.010
no	52(24.9)	157(75.1)	

^1^ Hinduism, Christianity, Buddhism; ^2^ primary incomplete = completed grade 1–4; ^3^ primary or secondary incomplete = completed grade 5–9; ^4^ secondary or higher = completed grade 10 or higher.

**Table 4:** Factors associated with women’s willingness to provide digital fingerprints (N = 458).

	Crude			Adjusted		
	OR	*P*	95% CI	OR	*P*	95% CI
Participant’s Characteristics						
Age (years)						
15–19	1.00			1.00		
20–24	1.15	0.697	0.57–2.32	0.79	0.538	0.36–1.69
25–29	1.77	0.134	0.84–3.73	1.26	0.590	0.55–2.90
30–34	1.01	0.988	0.46–2.22	0.91	0.843	0.37–2.27
35+	1.05	0.922	0.40–2.78	1.00	0.999	0.33–3.01
Level of Education						
no education	1.00			1.00		
primary incomplete ^1^	1.41	0.322	0.71–2.81	1.01	0.980	0.47–2.15
primary or secondary incomplete ^2^	1.62	0.115	0.89–2.95	1.28	0.497	0.63–2.62
secondary or higher ^3^	4.88	0.015	1.36–17.48	2.78	0.172	0.64–12.07
Involved in Income-Generating Activities						
no	1.00			1.00		
yes	0.63	0.112	0.35–1.11	1.61	0.139	0.86–3.04
Husband Characteristics						
Level of Education						
no education	1.00			1.00		
primary incomplete ^1^	1.34	0.419	0.66–2.70	1.06	0.880	0.50–2.26
primary or secondary incomplete ^2^	1.52	0.137	0.87–2.64	1.19	0.610	0.62–2.29
secondary or higher ^3^	3.37	0.020	1.21–9.34	1.75	0.353	0.54–5.67
occupation						
labourer	1.00			dropped from final model		
business	1.36	0.289	0.77–2.42			
regular job	1.38	0.274	0.78–2.44			
other	0.71	0.585	0.21–2.39			
Household Characteristics						
Sex of Household Head						
female	1.00			1.00		
male	3.17	0.016	1.23–8.12	2.65	0.062	0.95–7.34
Household Size						
>4	1.00			Dropped from final model		
≤4	1.26	0.332	0.79–2.02			
Type of Family						
extended	1.00			1.00		
nuclear	2.14	0.006	1.25–3.68	2.83	0.001	1.52–5.24
Wealth Status						
low	1.00			1.00		
moderate	1.42	0.264	0.77–2.61	1.38	0.333	0.72–2.68
high	3.57	0.000	2.07–6.15	2.96	0.000	1.63–5.39
Ownership of Mobile Phone						
no	1.00			1.00		
yes	1.84	0.010	1.15–2.93	1.42	0.191	0.84–2.40

^1^ Primary incomplete = completed grade 1–4; ^2^ primary or secondary incomplete = completed grade 5–9; ^3^ secondary or higher = completed grade 10 or higher.

## Data Availability

The data are available upon reasonable request from the corresponding author.

## References

[1] Evans R.S. (2016). Electronic health records: Then, now, and in the future. Yearb. Med. Inform..

[2] White E.B., Meyer A.J., Ggita J.M., Babirye D., Mark D., Ayakaka I., Haberer J.E., Katamba A., Armstrong-Hough M., Davis J.L. (2018). Feasibility, acceptability, and adoption of digital fingerprinting during contact investigation for tuberculosis in Kampala, Uganda: A parallel-convergent mixed-methods analysis. J. Med. Internet Res..

[3] Beck E.J., Shields J.M., Tanna G., Henning G., De Vega I., Andrews G., Boucher P., Benting L., Garcia-Calleja J.M., Cutler J. (2018). Developing and implementing national health identifiers in resource limited countries: Why, what, who, when and how?. Glob. Health Action.

[4] SonLa Study Group (2007). Using a fingerprint recognition system in a vaccine trial to avoid misclassification. Bull. World Health Organ..

[5] Tyagi N.K., Prasad J.B. (2020). Electronic Health Records in Health and Disease. Soc. Sci. Spectr..

[6] Marshall R., Rahman S. (2013). Internal migration in Bangladesh: Character, drivers and policy issues. United Nations Development Programme (UNDP).

[7] Razzaque A., Clair K., Chin B., Islam M.Z., Mia M.N., Chowdhury R., Mustafa A.H.M.G., Kuhn R. (2020). Association of time since migration from rural to urban slums and maternal and child outcomes: Dhaka (north and south) and Gazipur City corporations. J. Urban Health.

[8] Zulu E.M., Beguy D., Ezeh A.C., Bocquier P., Madise N.J., Cleland J., Falkingham J. (2011). Overview of migration, poverty and health dynamics in Nairobi City’s slum settlements. J. Urban Health.

[9] Bangladesh Bureau of Statistics (2015). Census of Slum Areas and Floating Population 2014.

[10] International Centre for Diarrhoeal Disease Research Baseline Population and Socioeconomic Census Slums of Dhaka (North and South) and Gazipur City Corporations, 2015–16. http://uphcp.gov.bd/cmsfiles/files/Baseline-Population%20and%20Socioeconomic%20Census.pdf.

[11] Mistry S.K., Akter F., Yadav U.N., Hossain M.B., Sichel A., Labrique A.B., Storisteanu D.M.L. (2021). Factors associated with mobile phone usage to access maternal and child healthcare among women of urban slums in Dhaka, Bangladesh: A cross-sectional study. BMJ Open.

[12] Jain A.K. (2007). Biometric recognition. Nature.

[13] Jain A.K., Bolle R., Pankanti S. (2006). Biometrics: Personal Identification in Networked Society.

[14] Joshi M., Mazumdar B., Dey S. (2020). A comprehensive security analysis of match-in-database fingerprint biometric system. Pattern Recognit. Lett..

[15] Unar J.A., Seng W.C., Abbasi A. (2014). A review of biometric technology along with trends and prospects. Pattern Recognit..

[16] Wall K.M., Kilembe W., Inambao M., Chen Y.N., McHoongo M., Kimaru L., Hammond Y.T., Sharkey T., Malama K., Fulton T.R. (2015). Implementation of an electronic fingerprint-linked data collection system: A feasibility and acceptability study among Zambian female sex workers. Glob. Health.

[17] Serwaa-Bonsu A., Herbst A., Reniers G., Ijaa W., Clark B., Kabudula C., Sankoh O. (2010). First experiences in the implementation of biometric technology to link data from Health and Demographic Surveillance Systems with health facility data. Glob. Health Action.

[18] Van Heerden A., Harris D.M., van Rooyen H., Barnabas R.V., Ramanathan N., Ngcobo N., Mpiyakhe Z., Comulada W.S. (2017). Perceived mHealth barriers and benefits for home-based HIV testing and counseling and other care: Qualitative findings from health officials, community health workers, and persons living with HIV in South Africa. Soc. Sci. Med..

[19] Choudhury N., Moran A.C., Alam M.A., Ahsan K.Z., Rashid S.F., Streatfield P.K. (2012). Beliefs and practices during pregnancy and childbirth in urban slums of Dhaka, Bangladesh. BMC Public Health.

[20] Storisteanu D.M.L., Norman T.L., Grigore A., Norman T.L. (2015). Biometric fingerprint system to enable rapid and accurate identification of beneficiaries. Glob. Health: Sci. Pract..

[21] American National Standard for Information Systems (1993). Data Format for the Interchange of Fingerprint Information, Doc# ANSI/NIST-CSL 1-1993.

[22] Jain A.K., Hong L., Pankanti S., Bolle R. (1997). An identity-authentication system using fingerprints. Proc. IEEE.

[23] Labrique A.B., Sikder S.S., Mehara S., Wu L., Huq R., Ali H., Christian P., Westr K. (2012). Mobile phone ownership and widespread mHealth use in 168,231 women of reproductive age in rural Bangladesh. J. Mob. Technol. Med..

[24] Bishwajit G., Hoque M.R., Yaya S. (2017). Disparities in the use of mobile phone for seeking childbirth services among women in the urban areas: Bangladesh Urban Health Survey. BMC Med. Inform. Decis. Mak..

[25] Ghose B., Feng D., Tang S., Yaya S., He Z., Udenigwe O., Ghosh S., Feng Z. (2017). Women’s decision-making autonomy and utilisation of maternal healthcare services: Results from the Bangladesh Demographic and Health Survey. BMJ Open.

[26] Khatun F., Heywood A.E., Hanifi S.M.A., Rahman M.S., Ray P.K., Liaw S.-T., Bhuiya A. (2017). Gender differentials in readiness and use of mHealth services in a rural area of Bangladesh. BMC Health Serv. Res..

[27] Johnson R., Wichern D. (2007). Multivariate Analysis.

[28] Rutstein S. (2015). Steps to Constructing the New DHS Wealth Index.

[29] Agresti A. (2002). Building and applying logistic regression models. Categ. Data Anal..

[30] Swedish International Development Agency (2010). Reality Check Bangladesh 2009-Listening to Poor People’s Realities About Primary Healthcare and Primary Education—Year 3.

[31] Senarath U., Gunawardena N.S. (2009). Women’s autonomy in decision making for health care in South Asia. Asia Pac. J. Public Health.

[32] Ahmed S.I., Haque M.R., Guha S., Rifat M.R., Dell N. Privacy, security, and surveillance in the global south: A study of biometric mobile SIM registration in Bangladesh. Proceedings of the 2017 CHI Conference on Human Factors in Computing Systems.

[33] Storisteanu D.M.L., Norman T.L., Grigore A., Labrique A.B. (2016). Can biometrics beat the developing world’s challenges?. Biom. Technol. Today.

[34] Mpembeni R.N.M., Kakoko D.C.V., Aasen H.S., Helland I. (2019). Realizing women s right to maternal health: A study of awareness of rights and utilization of maternal health services among reproductive age women in two rural districts in Tanzania. PLoS ONE.

[35] McKinley C.E., Liddell J., Lilly J. (2021). All Work and No Play: Indigenous Women “Pulling the Weight” in Home Life. Soc. Serv. Rev..

[36] Nguyen P.H., Frongillo E.A., Sanghvi T., Wable G., Mahmud Z., Tran L.M., Aktar B., Afsana K., Alayon S., Ruel M.T. (2018). Engagement of husbands in a maternal nutrition program substantially contributed to greater intake of micronutrient supplements and dietary diversity during pregnancy: Results of a cluster-randomized program evaluation in Bangladesh. J. Nutr..

[37] Aubel J. (2010). The Roles and Influence of Grandmothers and Men.

[38] Luke N., Xu H., Thampi B.V. (2014). Husbands’ participation in housework and child care in India. J. Marriage Fam..

[39] Karim R., Lindberg L., Wamala S., Emmelin M. (2018). Men’s perceptions of Women’s participation in development initiatives in rural Bangladesh. Am. J. Mens Health.

